# Occurrence of *Blastocystis* in Patients with *Clostridioides difficile* Infection

**DOI:** 10.3390/pathogens9040283

**Published:** 2020-04-14

**Authors:** Laura Vega, Giovanny Herrera, Marina Muñoz, Manuel Alfonso Patarroyo, Juan David Ramírez

**Affiliations:** 1 Grupo de Investigaciones Microbiológicas de la Universidad del Rosario (GIMUR), Departamento de Biología, Facultad de Ciencias Naturales, Universidad del Rosario, Bogotá 110221, Colombia; laurac.vega@urosario.edu.co (L.V.); giovannya.herrera@urosario.edu.co (G.H.); claudia.munoz@urosario.edu.co (M.M.); 2 Molecular Biology and Immunology Department, Fundación Instituto de Inmunología de Colombia (FIDIC), Bogotá 110221, Colombia; mapatarr.fidic@gmail.com; 3 School of Medicine and Health Sciences, Universidad del Rosario, Bogotá 110221, Colombia

**Keywords:** *C. difficile*, *Blastocystis*, dysbiosis, toxigenic *C. difficile*, community onset, healthcare facility onset

## Abstract

*Clostridiodes difficile* comprises a public-health threat that has been understudied in Colombia. Hypervirulent strains of *C. difficile* harbor multiple toxins, can be easily spread, and can have their onset of disease within healthcare facilities (HCFO) and the community (CO). Studies have shown that a disrupted microbiota (e.g., dysbiosis) may allow *C. difficile* infection (CDI). It has been suggested that dysbiosis prevents colonization by the anaerobic eukaryote *Blastocystis*, possibly due to an increase in luminal oxygen tension. No study has found co-occurrence of CDI and *Blastocystis*. Therefore, we aimed to determine the frequencies of *C. difficile* and *Blastocystis* infection/colonization in 220 diarrheal fecal samples. Molecular detection by PCR for both microorganisms was performed, with descriptive analyses of four variables (CDI detection, determination of *C. difficile* toxigenic profiles, *Blastocystis* detection, and patient site of onset). We demonstrate a significant association between the presence of *Blastocystis* and CDI, with coinfection found in 61 patients, and show a high frequency of CDI among both HCFO and CO groups. Our results of coinfection frequencies could support hypotheses that suggest *Blastocystis* can adapt to dysbiosis and oxidative stress. Further, the presence of toxigenic *C. difficile* occurring outside healthcare facilities shown here raises the alarm for community wide spread.

## 1. Introduction

Studies focused on the composition of the intestinal microbiota have indicated that some of its members may have a modulating effect on the diversity of other populations of microorganisms [1]. This is the case of *Clostridioides difficile*, which may be a negative modulator of the microbiota [2], which means that it can decrease the diversity of other groups of bacteria in the gut. For example, some authors have pointed out that *C. difficile* infection (CDI) is related to the decrease in beneficial bacteria while increasing the abundance of bacteria belonging to the Enterobacteriaceae family or other groups of bacterial pathogens [3]. Furthermore, some strains of *C. difficile* have importance in public health due to their virulence factors: toxins and mobile genetic elements (MGEs) [4]. Toxins A and B are encoded by the pathogenicity locus (*PaLoc*), which comprises five genes (*tcdA*, *tcdB*, *tcdR*, *tcdC*, and *tcdE*), while binary toxins (CdtA and CdtB) are encoded by *CdtLoc*. Likewise, MGEs of *C. difficile* are associated with resistance to some antibiotics, and sometimes, they can be transferred from other species of bacteria to strains of *C. difficile*. Hypervirulent strains are also important in public health since they can be easily spread and have aggressive clinical features [4].

*Blastocystis* is an anaerobic eukaryote that can be found in a wide range of hosts (humans, birds, primates, reptiles, etc.). This eukaryote exhibits a high genetic diversity and, thus, is classified in at least 17 subtypes, of which the distribution varies geographically [5,6]. *Blastocystis* may have a modulatory effect upon some members of the microbiota. However, due to its high genetic diversity, the variation of its subtypes distribution and the different results obtained when studying its modulatory effect on the microbiota has led to the difficulty of considering this eukaryote as a commensal of the gut microbiota or as a pathogen of the same. As a result of these controversies, the effect of *Blastocystis* colonization in the host health is still under study [7].

The microbiota-modulating effect of *Blastocystis* is still under study because microbiome studies in *Blastocystis* show variable results; some of them show that *Blastocystis* is associated with a healthy microbiota, while in others, it may be linked to dysbiosis and gastrointestinal diseases [7]. For instance, the in vivo and in vitro study carried by Yason et al. demonstrated that the presence of *Blastocystis* subtype 7 reduces the populations of beneficial bacteria in the gut microbiota while increasing populations of enterobacteria [8]. In addition, Nourrisson et al. showed that the abundance of beneficial bacteria, such as *Bifidobacterium* are reduced in patients with colonization by *Blastocystis* and inflammatory bowel disease (IBD) [9]. On the contrary, other studies have shown that the presence of *Blastocystis* in gut microbiota increases the abundance of some groups of beneficial bacteria (e.g., Ruminococcaceae, Lachnospiraceae, Clostridiaceae, etc.) [10,11,12].

Dysbiosis can be defined as changes in the communities of the microbiota that lead to a state of disease [13,14]. For instance, dysbiosis may be a common scenario in cases of diarrhea associated with the extensive use of antibiotics, which in turn allows *C. difficile* infection in the intestine [15]. However, it has been suggested that dysbiosis scenarios can prevent colonization by *Blastocystis* given that there is an increase in luminal oxygen concentrations postulated in dysbiosis [16]. Nonetheless, *Blastocystis* may have some mechanisms that allow it to respond to the oxidative stress of dysbiosis. Currently, no studies have shown the incidence of *C. difficile* and *Blastocystis* coinfection worldwide. Therefore, this work focused on determining the frequencies of *C. difficile* and *Blastocystis* infection/colonization in 220 fecal samples of Colombian patients with diarrhea, belonging to community-onset (CO) origin and healthcare facility-onset (HCFO) origin.

## 2. Results and Discussion

### 2.1. Descriptive Analyses of the Population and the Four Outcome Groups

**Figure 1:**
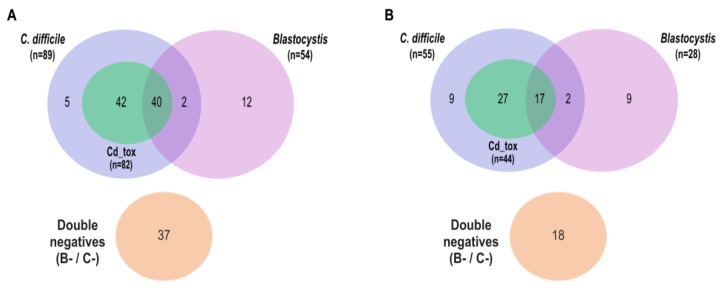
Infection frequencies by *Blastocystis* and *C. difficile* within onset groups: (**A**) Number of community-onset (CO) patients with *C. difficile* infection (CDI), positive toxigenic *C. difficile* (Cd_tox), and presence of *Blastocystis* and patients negative for both micoorganisms. (**B**) Number of healthcare facility-onset (HCFO) patients with *C. difficile* infection (CDI), positive toxigenic *C. difficile* (Cd_tox), and presence of *Blastocystis* and patients negative for both micoorganisms.

Descriptive analysis showed that 37.3% (n = 82) of the total population (n = 220) had *Blastocystis*. Markers of *C. difficile* were found in 65.4% (n = 144) of the total population, where 87.5% (n = 126) of these patients had *C. difficile* toxins. Also, most of the patients with *Blastocystis* or *C. difficile* or with some *C. difficile* toxin belonged to CO (Figure 1A,B). In the case of CO (n = 138), we found 89 patients with CDI, where 82 of these patients had a positive toxigenic *C. difficile* (Cd_tox); also, 40 patients belonging to this onset group presented three variables at the same time (CDI, positive Cd_tox, and presence of *Blastocystis*). Additionally within CO, 37 patients were negative for both study microorganisms (*Blastocystis* and *C. difficile*) (Figure 1A). Within HCFO (n = 82), we found 55 patients with CDI, where 44 had a positive toxigenic *C. difficile* (Cd_tox); only 17 patients belonging to this onset group presented the three variables mentioned above. Only 18 patients within HCFO were negative for both microorganisms (Figure 1B).

The sizes of each of our 4 outcome groups were as follows: 61 patients belonged to B+/C+, 55 patients belonged to B−/C−, 21 patients belonged to B+/C−, and 83 patients belonged to the group B−/C+. In Table 1 the distribution of CDI (toxins positive or negative) and *Blastocystis* among onset groups is detailed, where only a quarter of each population is free of the two microorganisms, and it is evident that most of the patients with CDI are toxin positive for both onset groups. Although for the group B+/C+ there is an increase of 8.3% in toxigenic *C. difficile* within CO in comparison to HCFO, the frequencies of toxigenic *C. difficile* are nearly identical among CO and HCFO of the group B−C+ (30.4% and 32.9%). Also, the results point out an excess of toxigenic *C. difficile* among all positive groups that belong to CO in contrast to HCFO (59.4% vs. 53.6%). A noticeable fact that jumps out is the excess of non-toxigenic *C. difficile* among HCFO, where most of these strains belong to the B−/C+ group (Table 1). In summary, it is important to highlight that, in all of the mentioned outcome groups, the distribution of cases based on CDI and/or *Blastocystis* status is similar among CO and HCFO.

The high rates of CDI both in CO and HCFO are in concordance with the studies that have reported similar results for the region. The study performed by Forero et al. (2019) in Colombia showed a high frequency of *C. difficile* infection in both onset groups, where 64.5% of CO patients and 67.1% of HCFO patients had CDI [17]. However, there are only a few studies in Colombia about the prevalence of CDI considering the onset groups mentioned herein, especially studies focusing on CDI within community onset. In the case of Latin America, an underestimation of CDI has been done, maybe because of the low standardization of methods for *C. difficile* detection and because of the lack of supplies in many laboratories [18]. Despite this fact, many studies performed in some countries such as Argentina, Brazil, Puerto Rico, and Chile have focused on the determination of CDI prevalence, principally within healthcare centers. Even though these studies focus on the healthcare center level, some of them highlight the importance of community-acquired *C. difficile* [19].

### 2.2. Frequencies of Coinfection Suggest a Possible Adaptation of Blastocystis to Oxidative Stress

The outcome group of greatest interest is the one positive for both microorganisms (B+/C+) since it has been suggested that, in the scenarios of dybiosis, there should be no colonization by *Blastocystis* because high concentrations of oxygen could affect *Blastocystis* survival [16]. Nevertheless, the results exposed here show the presence of *Blastocystis* and *C. difficile* in 61 patients out of 220. Also, a statistically significant association was obtained between the presence of *Blastocystis* and CDI (*p* = 0.032, Odds Ratio = 1.92). The present study is merely descriptive, and its limitations do not allow making conjectures about mechanisms that *Blastocystis* might be implementing to adapt to this environment that is not conducive to its survival, since these factors were not evaluated in this study. However, our results could support some hypothesis that have been proposed by other authors.

A previous study proposed a mechanism in which *Blastocystis* reduces the induced oxidative stress using its alternative oxidase system (AOX) [20], which may allow *Blastocystis* colonization to be successful even in environments where it is believed that its survival could be affected. Some studies not conducted in Latin America have found a low prevalence of *Blastocystis* in scenarios where there are alterations in the microbiota (such as those that may occur in inflammatory disease or Crohn’s disease) when compared with a healthy population [3,21]. In the case of Colombia, cases of coinfection of *Blastocystis* with other intestinal protists have been reported, and there has been suggested zoonotic transmission of some subtypes of *Blastocystis* [22,23].

Nevertheless, the results showed herein could suggest that *Blastocystis* may have the ability to adapt to other than ideal conditions (anaerobiosis). One hypothesis suggested by Laforest-Lapointe and Arrieta is that *Blastocystis* may have the ability to predate over some bacterial taxa within the disrupted microbiota [24], thereby promoting its survival under high oxygen concentrations. However, further studies are needed to provide additional information about the possible ecological interaction between *Blastocystis* and other members of the microbiota. Also, future approaches are required to elucidate the mechanisms that *Blastocystis* can use to adapt to a scenario of dysbiosis.

The infection frequencies shown in this work could suggest a possible adaptation of *Blastocystis* to a scenario of dysbiosis when there is also an infection by *C. difficile* and that had not been reported in Colombia or Latin America. Even though we found an association between CDI and *Blastocystis*, it is possible that there is an intermediate bacterial taxa in the microbiome mediating this association. In order to understand more deeply the role of *Blastocystis* in this scenario of dysbiosis, circulating subtypes must be identified within patient samples. Likewise, a descriptive analysis of the composition of each patient’s microbiota would help to completely elucidate the modulating role of this eukaryote on the members of the bacterial microbiome, particularly in those cases of coinfection with *C. difficile*.

### 2.3. Excess of Non-Toxigenic C. difficile among HCFO Patients

Surprisingly, toxigenic *C. difficile* was found equally among CO and HCFO, despite the expectation that it would be more common in the HCFO group. The latter group is expected to have more of the principal risk factor for CDI, alterations of the gut microbiota due to the extensive use of antibiotics and other medical treatments, as well as increased exposure to other patients with *C. difficile* due to prolonged hospitalization of the patients [25]. Indeed, an excess of non-toxigenic *C. difficile* was observed among HCFO patients in contrast to CO patients, especially those belonging to the B−/C+ group (Table 1). The previous result suggests that non-toxigenic *C. difficile* can be prevalent within healthcare facilities, where they could be clonally spread since these strains may be transformed into toxigenic strains by acquiring toxin genes, thus serving as potential pathogens.

On the other hand, a slight increment in the rate of toxigenic *C. difficile* among the CO group was identified in contrast with the HCFO group; however, this difference was not statistically significant. Even though CO has lower exposure to risk factors for CDI, in the last years, there has been an increase of CDI within CO patients [26], including the presence of some hypervirulent strains of *C. difficile* [27]. Our results support what was reported by Gupta and Khanna, where a study conducted in Cleveland found that more than half of asymptomatic residents were carriers of a toxigenic strain of *C. difficile* [28]. Additionally, in Colombia, Muñoz et al. reported a patient whose CDI had been acquired within the CO after antibiotic use that carried toxin-encoding genes and loci associated with antibiotic resistance [27]. In general, these findings support the hypothesis that CO patients can contribute to the transmission of *C. difficile* toxins [2,28].

Since CDI studies commonly use samples belonging to healthcare centers, the prevalence of CDI within CO patients and strain profiles of *C. difficile* within this onset group are less studied [27]. Some studies proposed that CDI in CO patients could arise due to antibiotic exposure and the use of other outpatient medications [29]. In this study, clinical data of the patients was not available, including prior antibiotic use or recent hospitalizations, which might have clarified how CDI within the CO group could have arisen as an emerging transmission scenario. Studies have highlighted the ability of some *C. difficile* CO strains to acquire virulence factors (e.g., toxin-encoding genes) [30] via horizontal gene transfer to the non-toxigenic strains [31]. This high level of genome plasticity is supported by previous studies of our group, where strains circulating in CO were characterized finding rearrangements in these genes [27,30]. Finally, this high frequency of CDI could be explained by an increased clonal spread, in which some limited sequence types (STs) have been detected within the community. However, there is a lack of studies focused on molecular or genomic epidemiology that can help to elucidate population structure and transmission dynamics of *C. difficile* in Colombia [30,32]. Future studies should consider the implications of CDI in CO.

## 3. Materials and Methods

Only patients with diarrhea (the main manifestation of CDI) were included in this study, since it would allow a greater probability of identifying patients with alterations in intestinal homeostasis, including *C. difficile* infection. Thus, 220 previous stool samples were employed, and the procedures of collection and subsequent DNA extraction of these samples were conducted as reported in Muñoz et al. (2018) [32]. Briefly, CDI was determined by different approaches including in vitro culture and molecular methods: conventional PCR targeting *16S-rRNA* and *gdh* and quantitative PCR targeting *16S-rRNA*. Afterward, toxigenic *C. difficile* of samples positive for any CDI molecular detection were determined by conventional PCR targeting six molecular markers located within *PaLoc* and *CdtLoc*. The amplification of lok1/lok3 markers flanquing the *PaLoc* was performed to determine those *C. difficile* strains without *PaLoc* [32].

The purpose of targeting more than one molecular marker in the previous study is because of its usefulness for increasing the sensibility and specificity in the schemes employed in the molecular epidemiology of pathogens, especially when the microorganism has a highly dynamic genome (e.g., *C. difficile*). The high genetic diversity of the markers can lead to an underestimation of their frequency when only one test is used [32]. Although we employed the same population as in Muñoz et al. (2018), the present study considered different selection criteria. Hence, the selection criteria used to determine positive samples (for CDI or Cd_tox) was when at least one positive result in any of the traditional molecular tests applied to DNA of the sample was obtained. Thus, in the case of toxigenic *C. difficile*, the presence of genes that encode for any of the toxins (toxins A or B, or binary toxin) determined a positive toxigenic *C. difficile*, since some studies show that binary toxins have cytopathic effects in vitro and exacerbate clinical outcomes in *C. difficile* patients [33,34]. Therefore, all toxigenic CDI samples had at least 2 molecular markers positive (*16S-rRNA* or *gdh* gene and at least 1 of the toxin genes).

The origin or onset groups of the patients (community-onset (CO) or healthcare facility-onset (HCFO)) was determined considering the guidelines of the Clinical Practice Guidelines for CDI in Adults [35]. The mentioned guideline defines three categories for patient classification, where two of them contemplate the community classification: community-onset, healthcare facility-associated disease (CO-HCFA), and community-associated CDI (CA-CDI) [35]. Despite this classification, for the present study, the categories that contemplate the community onset (CO-HCFA and CA-CDI) were grouped as one (CO). This grouping of community onset was performed given the lack of clinical data of CO patients, especially information about prior hospitalizations [32]. Hence, CO was defined when the patient attended the emergency room for different reasons and presented an episode of diarrhea during the 48 h following admission to a medical center. For the CO subjects, 85% of these patients attended the emergency room while 15% were referred for outpatient consult. On the other hand, an HCFO patient was defined when the episode of diarrhea developed after the third day following admission. HCFO also included patients from Intensive Care Unity (ICU), which constitutes a population with major risks for developing complications associated with CDI [32].

Likewise, *Blastocystis* presence in 220 stool samples was determined using a conventional PCR as reported elsewhere [36]. A database was constructed to couple the information corresponding to CDI, successful determination of toxigenic *C. difficile* (Cd_tox), presence of *Blastocystis*, and the patients’ onset group (CO or HCFO). Furthermore, the database allowed us to define 4 outcome groups: positive for *Blastocystis* and *C. difficile* (B+/C+), only positive for *C. difficile* (B−/C+), only positive for *Blastocystis* (B+/C−), and negative for both microorganisms (B−/C−). We constructed Venn diagrams to better depict the number of samples in the described groups, including data from Cd_tox that was also plotted. Descriptive analyses were performed to determine the frequencies of coinfection events in terms of percentage. Finally, X^2^ tests were performed to identify the associations between the four variables of interest (CDI, presence of *Blastocystis*, Cd_tox, and patients’ onset group). For the cases in which a significant association between two variables was obtained, an odds ratio (OR) was calculated considering only the CDI positive samples (n = 144) and taking HCFO as the onset group with increased risk (IC = 95%). The clinical importance of using only CDI positive samples is to know which percentage of these samples carry toxin genes and which of them are non-toxigenic. The statistical analyses were performed in STATA 12.0 (StataCorp LLC, College Station, TX, USA). A significance value *p* < 0.05 was fixed for all hypothesis tests.

## Figures and Tables

**Table 1:** Distrbution of CDI (toxins positive or negative) and *Blastocystis* among community-onset (CO) or healthcare facility-onset (HCFO).

	B+/C+ ^1^		B−/C+ ^1^		B−/C−	B+/C−
	Cd_tox Postitive	Cd_tox Negative	Cd_tox Postitive	Cd_tox Negative	_	_
CO ^2^ *n* = 138	29.0%	1.4%	30.4%	3.6%	26.8%	8.7%
HCFO ^2^ *n* = 82	20.7%	2.4%	32.9%	11.0%	22.0%	11.0%

^1^ These groups present an additional category, positive or negative toxigenic *C. difficile*, because these outcome groups are the only ones positive for CDI; ^2^ percentages were calculated considering the total samples for each onset type.
